# Medical Education—the Northwest Still in the Lead

**Published:** 1880-07

**Authors:** 


MEDICAL EDUCATION.—THE NORTHWEST STILL IN THE LEAD.

While the medical department of Harvard has been simply giving notice of an intention to add a *fourth year* to the period of medical study at a future time, which, however, was to be optional with the student, whether he should take it or not, the new St. Paul Medical College, Medical Department of Hamline University, boldly announces its adoption of a full graded course extending over *four years*, a full compliance with which is made obligatory upon all candidates for graduation. We quote from the annual announcement for 1880–81, as follows: "Instruction is given by lectures, recitations, and clinical teaching, distributed throughout the academic year, which commences on the first Tuesday in October and closes on the last Saturday in May, with a recess of ten days at Christmas.

"The course of study has been enlarged and extends over *four years*, and the examinations for a degree are divided into *four*, one to be held at the close of each year.

"All examinations, both in the regular and adjunct branches, must be passed at sometime previous to receiving the diploma.

"An examination on entrance, in the higher English branches is required."

Will the medical press of the whole country take as much pains to notice the bold and positive stand taken by the young school in Minnesota, as it has the simple declaration on the part of Harvard of an intention to do something in the future?

